# Transcriptomic characterization of maturing neurons from human neural stem cells across developmental time points

**DOI:** 10.1016/j.ibneur.2025.04.013

**Published:** 2025-04-17

**Authors:** Kimia Hosseini, Gaëtan Philippot, Sara B. Salomonsson, Andrea Cediel-Ulloa, Elnaz Gholizadeh, Robert Fredriksson

**Affiliations:** a Department of Pharmaceutical Bioscience, Uppsala University, Sweden; b Department of Organismal Biology, Uppsala University, Sweden

**Keywords:** Differentiation, Maturation, Neural stem cells, Neurodevelopment, In-vitro

## Abstract

Neurodevelopmental studies employing animal models encounter challenges due to interspecies differences and ethical concerns. Maturing neurons of human origin, undergoing several developmental stages, present a powerful alternative. In this study, human embryonic stem cell (H9 cell line) was differentiated into neural stem cells and subsequently matured into neurons over 30 days. Ion AmpliSeq™ was used for transcriptomic characterization of human stem cell-derived neurons at multiple time points. Data analysis revealed a progressive increase of markers associated with neuronal development and astrocyte markers, indicating the establishment of a co-culture accommodating both glial and neurons. Transcriptomic and pathway enrichment analysis also revealed a more pronounced GABAergic phenotype in the neurons, signifying their specialization toward this cell type. The findings confirm the robustness of these cells across different passages and demonstrate detailed progression through stages of development. The model is intended for neurodevelopmental applications and can be adapted to investigate how genetic modifications or exposure to chemicals, pharmaceuticals, and other environmental factors influence neurons and glial maturation.

## 1 Introduction

In the process of brain development, the central nervous system (CNS), which is recognized as the most complex organ (Hayes and Nowakowski, 1999), originates from a small number of neural stem cells (NSCs) lining the neural tube (Kriegstein and Alvarez-Buylla, 2009). NSCs were among the first types of cells generated from embryonic stem cells (ESCs) *in vitro*. Due to their unique capacity to propagate in culture over multiple passages and proliferate and differentiate into both neurons and glial cells, they are considered an attractive tool to explore cellular processes and can offer valuable insights into the mechanisms involved in neural induction and neurogenesis (Chuang et al., 2015, Oh et al., 2018). Extensive research and studies of these cells have led to the development of differentiation protocols to achieve better and more reproducible cultures for translational medicine and stem cell research (de Leeuw et al., 2020, Heikkilä et al., 2009)

Animal models have consistently played an important part in neurodevelopmental studies (Halliwell, 2018). However, they pose challenges including high maintenance costs, and ethical considerations related to the use of animals in laboratory settings (Baumans, 2004), and in general, the models are limited in their ability to unveil certain fundamental aspects of human brain development and diseases (Zhao and Bhattacharyya, 2018) These limitations are particularly pronounced in brain disorders, where the differences between humans and animal models are most significant (Zhao and Bhattacharyya, 2018). Given the ethical and practical challenges associated with obtaining fetal human brain tissue, the development, and use of alternative human-specific model systems that are both accessible and ethically sound are essential for advancing research in this field (Damianidou et al., 2022). *In vitro* tools, particularly human ESCs (hESCs) in general and human NSCs (hNSCs) for neurodevelopmental studies, are gaining increased interest as alternatives or complements to animal models. These models offer key advantages, particularly because ethical restrictions limit research on human embryos and fetuses, and studying development in utero is not feasible. Moreover, animal models often differ from human development due to evolutionary divergence and typically require genetic manipulations that can unintentionally impact other genes and molecular pathways (Telias and Ben-Yosef, 2014). Although completely replacing animal models with *in vitro* systems would be ideal, it may not be feasible soon. However, minimizing the use of laboratory animals should remain a key objective for *in vitro* studies.

To ensure the validity of the neurodevelopmental study, a comprehensively understood and characterized cell model is essential. Cellular characterization offers the possibility to define signaling pathways and molecular interactions. For example, it can help in the identification of specific cellular markers associated with different developmental stages (Li et al., 2017, van de Leemput et al., 2014). Further, characterization can show if the culture is undergoing normal development or if it is affected, based on the experimental design. This study aimed to characterize maturing neurons in an adherent monolayer culture (2D) undergoing developmental stages over 30 days. The 2D culture was chosen over the 3D culture due to a simpler protocol and lower cost of maintenance (Kapałczyńska et al., 2018). The goal was to evaluate the stage-specific changes in gene expression across multiple times during neuronal maturation from the stem cell stage, using the Ion AmpliSeq™ technique developed by Thermo Fisher Scientific. Ion AmpliSeq™ gene profiling enables the identification of robust changes in gene expression over time, providing valuable insights into the dynamics of gene regulation. It is a targeted and multiplexed PCR-based transcriptome technology, measuring the level of expression of more than 20,000 genes even from low quantities (1 ng) or even degraded RNA samples (Alessandrini et al., 2020). In cell differentiation experiments, where sample quantity may be limited, this approach proves to be particularly useful.

This *in vitro* model, employing human cells, provides thorough genetic characterization and identification of enriched pathways during development, offering valuable mechanistic insights. This makes it suitable for neurodevelopmental studies (Hosseini et al., 2024) and other applications requiring a detailed understanding of neuronal processes.

## 2 Materials and methods

### 2.1 Cell culture

**Fig. 1:**
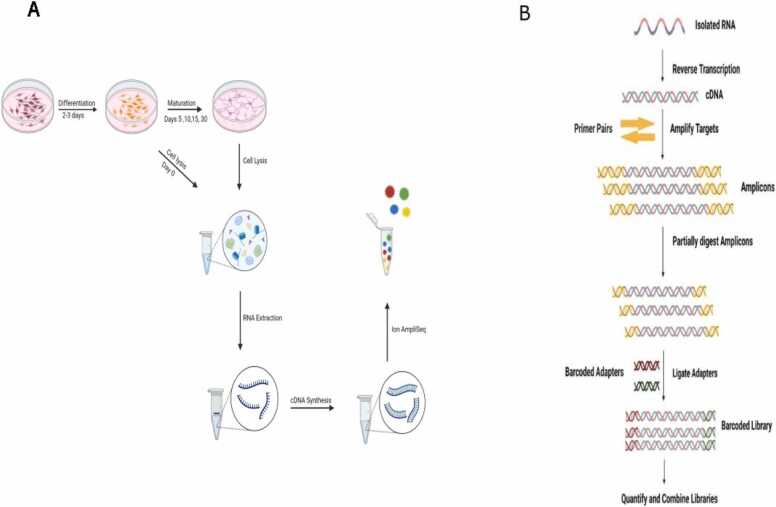
Schematic representation of the Ion AmpliSeq™ protocol and experimental setup diagram: A. Illustration of the experimental setup used for sample preparation, starting from hNSCs culture and progressing through various time points of maturation. B. Schematic workflow of Ion AmpliSeq™ library preparation (adapted from illustration by Thermo Fisher Scientific). Illustrations were created with Biorender.

The hNSCs were derived from hESCs (cell line WA09, alias H9, WiCell) as adherent culture using neural induction medium (Thermo Fisher Scientific Cat. No A1647801). The procedure is described in detail in our previous publication (Hosseini et al., 2020). For the downstream analysis of this paper, hNSCs were seeded at a density of 6 × 10^4^/cm^2^ onto double-coated 6-well plates. Initially, Poly-L-ornithine hydrobromide (PLO) (Sigma-Aldrich Cat. No. P4957) was applied as the first layer, and the plates were kept in the incubator for at least 1.5 hours. Subsequently, the culture plates were thoroughly washed two times with PBS and then coated with Laminin (Sigma Cat. No. L2020), diluted in Advanced™ DMEM⁄F-12 (Thermo Fisher Scientific Cat. No. 12634), for an additional 1.5 hours. The cells were seeded and maintained in a neural expansion medium consisting of 49 ml Advanced™ DMEM⁄F-12, 49 ml of Neurobasal® Medium, and 2 ml GIBCO neural induction supplement (Thermo Fisher Scientific Cat. No. A1647801) overnight at 37°C and 5 % CO_2_. The next day, the medium was replaced with STEMdiff™ Forebrain Neuron Differentiation Medium (Stem Cell Technology Cat. No. 08600), which was changed daily for 2-3 days until the cells reached 80 % confluency. At this stage, the neuronal progenitors were ready for final plating. The cells were harvested using StemPro® Accutase® (Thermo Fisher Scientific Cat. No. A1110501) and seeded onto PLO/Laminin coated culture plates at the required density, followed by incubation at 37°C and 5 % CO_2_ as specified in the STEMdiff™ Forebrain Neuron Maturation Kit (Stem Cell Technology Cat. No. 08605). Day 0, designed as the earliest time point, served as the reference control. At this stage, the neural stem cells were still in the neural forebrain differentiation medium but were 80 % confluent. Samples for this time point were collected just before the final plating and induction into the maturation phase. Upon transfer to the maturation medium, it was refreshed every 2–3 days with a fresh room-temperature medium. Subsequent samples were harvested for downstream analysis at desired time points (Fig. 1 A).

### 2.2 Immunofluorescence staining and microscopy

Cells were seeded at a density of 1.5 × 10^4^ cells/cm^2^ on coverslips previously coated with PLO and Laminin. Cells were later fixed using 4 % paraformaldehyde (PFA) at days 0, 5, 15, and 30 for antibody staining and further visualization. Class III β-tubulin (β-III-tub) (Abcam, ab18207 diluted, 1:200) was used as a primary antibody to stain the maturing neurons. On the second day, cells were washed 3x with PBS and incubated with the corresponding secondary antibody for 2 hours. Both primary and secondary antibodies were diluted in Supermix (200 ml TBS, 0.5 g gelatin, and 1 ml Triton X-100). As the last step, coverslips were mounted with ProLong Gold Antifade Mountant with DAPI (Thermo Fisher Scientific, Cat. No. P36931). DAPI was used as a nuclear counterstain. Images were acquired at the Scilifelab BioVis facility (Uppsala University, Sweden) using the confocal Zeiss ELYRA S.1 and Zen Black software (Zeiss, Oberkochen, Germany). The cells were imaged using a 40X objective lens, and the acquired TIFF images were analyzed with Cell Profiler version 3.1.9. For this, a pipeline was designed to identify primary objects (nuclei) and secondary objects (neurons) and to quantify the integrated intensity of each measured object.

### 2.3 RNA extraction

To prepare RNA samples, cells were seeded at a density of 4 × 10^4^ cells/cm^2^ on PLO/Laminin-coated four-well plates. At each time point, an Aurum ™ Total RNA Mini Kit (Bio-Rad Cat. No. 7326820) was used to extract DNA-free total RNA from lysed neurons, according to the manufacturer’s protocol. The extracted RNA from each well was eluted in 40 µL of nuclease-free water, and the material from two wells was pooled to obtain one sample. This pooling strategy aimed to maintain consistency between technical replicates while minimizing variability. Additionally, it's worth noting that for each time point, only two wells out of the four-well plate were used for seeding. Samples were then stored at −80⁰ for later analysis. Concentrations were measured using an ND-1000 spectrophotometer (NanoDrop Technologies), and subsequently, RNA integrity was controlled using an Agilent 2100 Bioanalyzer. RNA samples were collected from maturing neurons at five different time points as follows; day 0, 5,10, 15, and 30. In total, 4 to 5 different passages of hNSCs were used as independent replicates. The rationale for selecting these specific time points was to comprehensively capture the entire duration of the culture period. Gene expression during development may not exhibit significant changes overnight; therefore, choosing multiple time points helps to capture key stages from early maturation to the final day of culture. While more frequent sampling could provide additional insight, it was not feasible within the available limits.

### 2.4 Ion AmpliSeq™ library preparation

Ten ng of RNA was reverse transcribed according to the Ion AmpliSeq™ Transcriptome Human Gene Expression Kit Preparation protocol (Thermo Fisher). The cDNA was amplified using Ion AmpliSeq™ Transcriptome Human Gene Expression core panel (Thermo Fisher), and the primer sequences were then partially digested. Subsequently, adaptors (Ion P1 Adapter and Ion Xpress™ Barcode Adapter, Life Technologies) were ligated to the amplicons. Adaptor ligated amplicons were purified using Agencourt® AMPure® XP reagent (Beckman Coulter) and eluted in the amplification mix (Platinum® PCR SuperMix High Fidelity and Library Amplification Primer Mix, Thermo Fisher) and amplified. Size selection and purification were conducted using Agencourt® AMPure® XP reagent (Beckman Coulter). The amplicons were quantified using the Fragment Analyzer™ instrument (Advanced Analytical Technologies, INC.), with DNF-474 High Sensitivity NGS Fragment Analysis Kit (Advanced Analytical Technologies, INC.). Samples were then pooled, eight or fewer per pool, followed by emulsion PCR on the Ion Chef™ System using the Ion 550™ Kit-Chef (Thermo Fisher). The pooled samples were loaded on one Ion 550™ chip and sequenced on the Ion S5XL™ System using the Ion S5 Sequencing chemistry (200 bp read length, Thermo Fisher). The raw data was converted to the number of reads per transcript using an AmpliSeq RNA plugin (Illumina, San Diego, USA) for the Torrent Suite Software (Illumina, San Diego, USA) (Fig. 1B).

### 2.5 Transcriptomic analysis

Analysis of gene expression data was performed using R version 4.3.1 with the packages DESeq2 1.40.2 (Love et al., 2014), ggplot2 3.4.2 (Wickham, 2016), and Enhanced Volcano 1.18.0 (Kevin Blighe et al., 2023). The data was visualized by generating a principal component analysis (PCA) plot based on the top 100 genes driving the PCA using the ggplot2 package. Pathway enrichment analysis (PEA) and cell type specificity using Enrichr-KG (Evangelista et al., 2023), including the libraries Reactome (Fabregat et al., 2017), KEGG (Kanehisa, 2000), and Human Gene Atlas (Su et al., 2002), were further performed to evaluate the top 100 genes driving the PCA. Differential expression analysis was performed in pairs between all-time points, using the DESeq2 package (adjusted p-value (FDR) < 0.001). The distribution of the data for each comparison was further evaluated with volcano plots. Heatmaps for graphical representation of data were created using Genesis version 1.8.1 (Sturn et al., 2002). Individual markers for different stages of neuronal cells were further assessed separately. This was done using raw counts from the transcriptomic analysis, normalized with the median-of-ratios method in DESeq2.

### 2.6 Statistical analysis

Each NSC passage that was induced to differentiation and maturation was considered 1 independent replicate. Statistical analysis of gene expression data from Ion AmpliSeq™ was performed on count data from 4 to 5 independent replicates (n = 4–5), which were normalized using the median-of-ratios method in DESeq2.

**Fig. 2:**
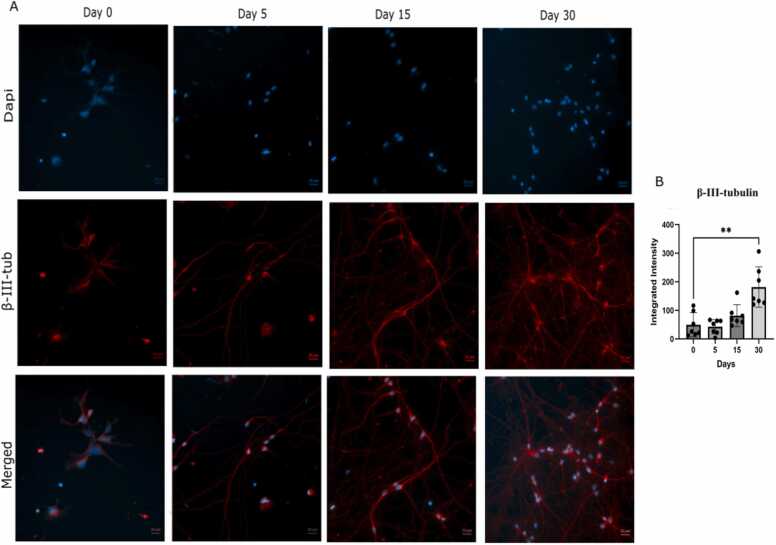
Visualization of the neurons using immunocytochemistry at various time points. A. Representative images of cells stained with the neuronal marker β-III-tubulin and counterstained with DAPI. All images include a scale bar of 20 µm. B. Quantification of the integrated β-III-tubulin intensity over time, showing increased expression during maturation. Data are presented as mean ± SEM (n = 7). Statistically significant differences are indicated by asterisks ** p < 0.01

For Fig. 2, Fig. 5, and supplementary, the normality of the data was assessed with the Shapiro-Wilk test. If normal distribution was indicated, one-way ANOVA followed by Dunnett's multiple comparisons test was used. If the data did not meet normality assumptions, a non-parametric Kruskal-Wallis test followed by Dunn’s multiple comparisons test was employed. Graphs were plotted using mean values with error bars representing the standard error of the mean (SEM) in GraphPad Prism version 10.2.2 (GraphPad Software, www.graphpad.com). Significant differences between the mean values of Days 5, 10, 15, and 30 relative to Day 0 were evaluated and were defined as follows: *p < 0.05, **p < 0.01, ***p < 0.001, and *** *p < 0.0001.

## 3 Results

### 3.1 Morphological visualization by immunocytochemistry showed an increase in the protein level of β-III-tubulin over time

To visualize the culture, cells were stained with the neuronal marker β-III-tubulin (Fig. 2 A), and signal intensity was quantified using CellProfiler version 3.1.9 (Fig. 2B). The β-III-tubulin integrated intensity ratio showed a progressive increase over time, with a statistically significant elevation at day 30, indicating neuronal identity and progressive maturation.

### 3.2 Transcriptome analysis by Ion AmpliSeq™ reveals dynamic gene expression changes over time during the maturation of neurons derived from hNSCs

**Fig. 3:**
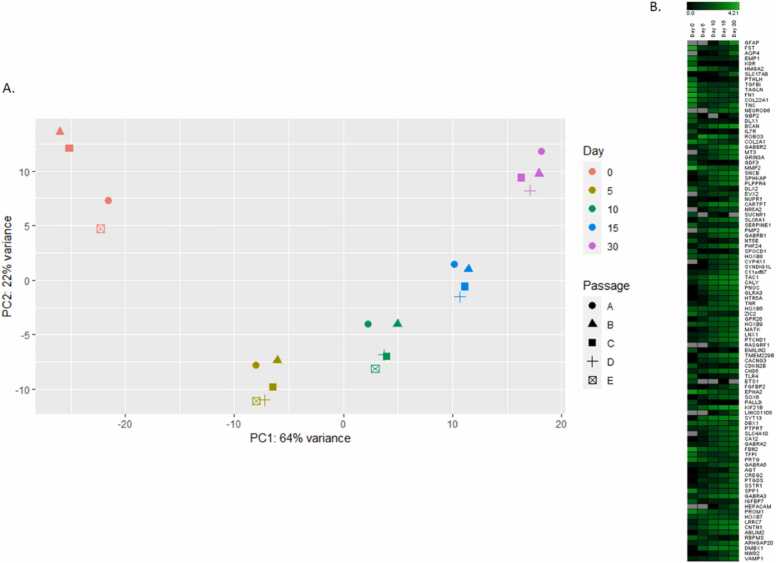
PCA showing gene expression patterns: A. PCA plot based on the top 100 genes driving the variance. The samples are clustered in relation to days of maturation, with the different passages in proximity to one another. B. Heatmap showing the expression level by maturation day of the top 100 genes driving the PCA, normalized by the median-of-ratios method in DESeq2.

**Fig. 4:**
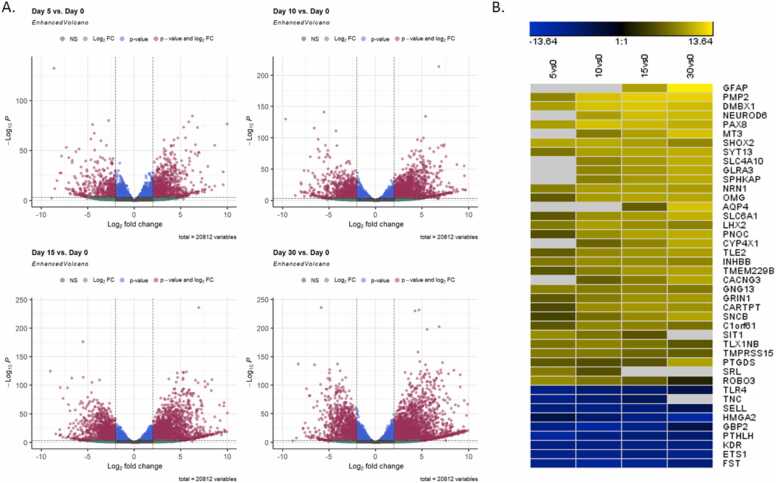
Distribution of differentially expressed genes. A. Volcano plots showing dispersion of all genes at each time point versus day 0. Differentially expressed genes indicate a similar distribution of up and downregulated genes. Limits are drawn at adjusted p-value 0.001 and log2 fold change 2.0. B. Heatmap showing expression levels of the top 20 differentially expressed at each time point versus day 0. The color gradient represents expression changes from blue to yellow. Grey sections indicate values that were not differentially expressed compared to day 0.

The Ion AmpliSeq™ method was used to perform transcriptome RNA sequencing on cells from five different time points to elucidate various stages of neural development. PCA was conducted based on the top 100 genes driving the variance of all samples (Fig. 3A), revealing that cells clustered primarily by maturation day (64 % of the variance). A graphical representation of the top 100 genes is shown in Fig. 3B. Differential expression analysis of genes was performed in pairs with an FDR cutoff of 0.001. For further analysis, absolute Log2FC was limited to > 2.0, with the following result: 1246 genes for day 5 vs day 0 (839 upregulated, 407 downregulated); 2371 genes for day 10 vs day 0 (1459 upregulated, 912 downregulated); 2830 genes for day 15 vs day 0 (1790 upregulated, 1040 downregulated); 3483 genes for day 30 vs day 0 (2354 upregulated, 1129 downregulated); 304 genes for day 10 vs day 5 (226 upregulated, 78 downregulated); 1053 genes for day 15 vs day 5 (699 upregulated, 354 downregulated); 2051 genes for day 30 vs day 5 (1427 upregulated, 624 downregulated); 29 genes for day 15 vs day 10 (26 upregulated, 3 downregulated); 684 genes for day 30 vs day 10 (564 upregulated, 120 downregulated); 104 genes for day 30 vs day 15 (88 upregulated, 16 downregulated). Volcano plots illustrate an evenly distributed differential expression of up- and downregulated genes between different time points versus day 0 (Fig. 4A). The normalized gene count of the top 20 differentially expressed genes for each comparison was further compiled graphically in a heat map, illustrating a continuous up or downregulation over the time course of maturation. The compilation resulted in 42 genes due to several genes occurring in the top 20 in more than one comparison (Fig. 4B. The genes from this compilation that are associated with neuronal development or have a function in the nervous system are summarized in Table 1.

**Table 1:** Genes associated with neuronal development and CNS function. This table compiles the genes ranked among the top 20 in terms of differential expression that are shown in Fig. 4B and have association with neuronal development and CNS function. Each gene's specific role in processes such as nervous system development, migration, axogenesis, and synaptic plasticity is summarized within the table.

**Symbol**	**Gene Name**	**CNS Function**
NeuroD6	Neuronal Differentiation 6	Development of nervous system (Uittenbogaard et al., 2010)
NRN1	Neuritin 1	Neuronal migration (Zito et al., 2014)
C1orf61	Chromosome 1 open reading frame 61	Brain development and neuronal plasticity (Jeffrey et al., 2000)
LHX2	LIM Homeobox Protein 2	Development of nervous system (Hou et al., 2013) and Forebrain regulator (Chou and Tole, 2019)
DMBX1	Diencephalon/Mesencephalon Homeobox 1	Maturation of neuro-olivary projections (Schilling, 2024)
SHOX2	SHOX Homeobox 2	Vestibular neuron development (Laureano et al., 2022)
SLC4A10	Solute Carrier Family 4, Sodium Bicarbonate Transporter, Member 10	Modulation of short term plasticity in Hippocampus (Sinning and Liebmann, 2015)
ROBO3	Roundabout Guidance Receptor 3	Axogenesis (Pak et al., 2020) and Neuronal migration (Cariboni et al., 2012)
TNC	Tenascin C	Neuronal migration and synaptic plasticity (Šekeljić and Andjus, 2012)
GRIN1	Glutamate Receptor, Ionotropic, N-Methyl D-Aspartate 1	Synaptic plasticity (Sengar et al., 2019)
PTGDS	Prostaglandin D2 Synthase	Axogenesis (Upadhya et al., 2020)
SLC6A1	Solute Carrier Family 6 Member 1	GABA reuptake into presynaptic neurons (Mermer et al., 2021)
AQP4	Aquaporin-4	Synaptic plasticity and astrocyte marker (Vandebroek and Yasui, 2020)
GFAP	Glial Fibrillary Acidic Protein	Astroglial marker (Baba et al., 1997)

**Fig. 5:**
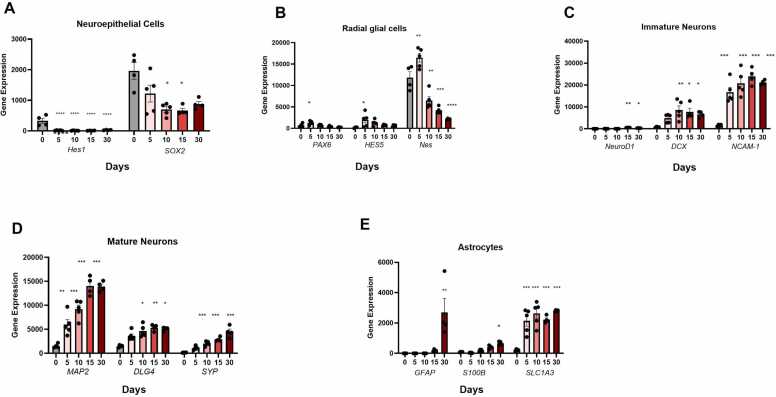
Expression level of selected neuronal and astrocyte markers related to different stages of neural development. A. Expression levels of neuroepithelial markers, showing a decrease over the culture duration. B. Radial glial markers show an initial increase and then a decrease in expression level. C. Immature neuronal markers show an increase in expression by day 10–15 and a slight decrease by day 30. D. Mature neuronal markers show either a continued increase after day 15 or plateauing. E. Astrocyte markers showed expression during the whole period except *GFAP*, which only emerged at day 15 and was significantly upregulated by day 30. Statistically significant results are indicated with asterisks (* p < 0.05, ** p < 0.01, *** p < 0.001, **** p < 0,0001).

To further assess the neuronal identity of the cell cultures, several of the classical markers of neural lineage were analyzed at different stages of maturation and are summarized in Fig. 5. Neuroepithelial makers Sox2 and Hes1 are downregulated as the culture time increases. Radial glial markers (*Pax 6, Hes 5*, and *Nes*) peak at day 5 but start to downregulate thereafter. Markers of immature neurons (*NeuroD1, DCX, and NCAM-1*) show upregulation, reaching their peak at day 10–15. Maturation markers (*MAP2, DLG4,* and *SYP*) exhibit upregulation, reaching their peak at day 15–30. Astrocyte markers (*SLC1A3, S100β*) show expression from the early stages and progressively increase over time. Conversely, *GFAP* shows a delayed emergence, with a significant increase observed by day 30. These findings highlight the dynamic nature of gene expression during the maturation of neuronal cells (Fig. 5). Due to the possible heterogeneity of the neurons in the culture, we selectively chose and investigated the expression of several neuronal markers to assess the extent of cellular diversity. GABAergic markers showed the highest expression, progressively increasing over time and aligning with unbiased analyses. Glutamatergic and then cholinergic markers were also present, though consistently at lower levels. Markers for dopaminergic and serotonergic neurons were either absent or expressed at very low levels, except *LMX1 B* (dopaminergic), which showed an initial increase followed by subsequent downregulation (supplementary Figure 1).

### 3.3 Pathway enrichment analysis shows neuronal-specific differentiation

**Fig. 6:**
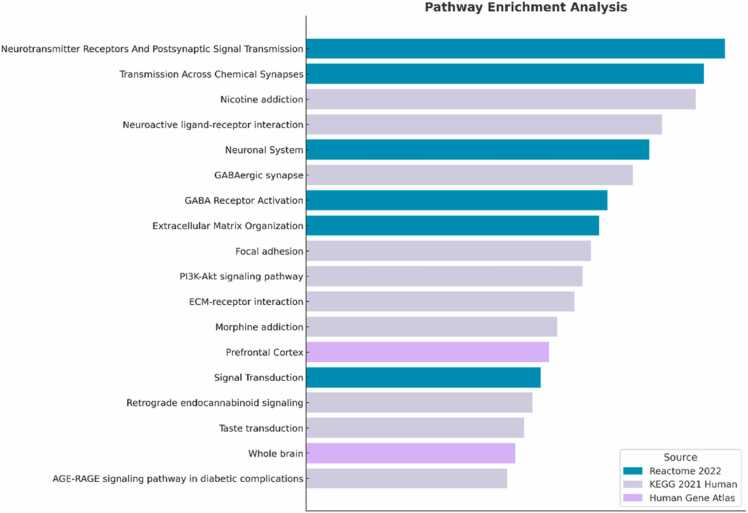
Visualization of enriched pathways and cell types. Enrichment analysis was performed on the top 100 genes driving the PCA. The bar graph shows the top enriched terms (q < 0.05) using the databases KEGG, Reactome, and Human Gene Atlas. The databases KEGG and Reactome provide enriched pathways, while the Human Gene Atlas provides cell types. A larger bar implies a lower q-value, indicating a more significant enrichment.

Enrichment analysis with Enrichr-KG was performed on the top 100 genes driving the PCA, using the datasets Human Gene Atlas, Reactome, and KEGG. All pathways and cell types with a q-value < 0.05 were considered (Fig. 6 and Table 2). Two cell types came up as significant

**Table 2:** Table showing results of pathway enrichment analysis with KEGG and Reactome datasets, and cell type evaluation with Human Gene atlas, using Enrichr-KG. The results with higher significance based on the corrected p-value (q-value) are at the top of the table. All 100 top genes driving the clustering in the PCA were included in the analysis. The listed genes are genes driving the enrichment of pathways or cell types (terms).

Term	Library	q-value	Genes
Neurotransmitter Receptors And Postsynaptic Signal Transmission R-HSA−112314	Reactome 2022	0.0000056239	CACNG3, GABRA3, GRIN3A, GABRA2, GLRA3, GABBR2, GABRA5, RASGRF1, LRRC7, GABRB1
Transmission Across Chemical Synapses R-HSA−112315	Reactome 2022	0.0000056239	GABRB1, GLRA3, GABBR2, GABRA5, RASGRF1, LRRC7, GABRA2, GABRA3, GRIN3A, CACNG3, SLC6A1
Nicotine addiction	KEGG 2021 Human	0.000005119	GABRA2, GRIN3A, GABRB1, GABRA5, SLC17A8, GABRA3
Neuroactive ligand-receptor interaction	KEGG 2021 Human	0.000062916	AGT, HTR5A, TAC1, GABRA2, GABBR2, SSTR1, GABRB1, GABRA3, GLRA3, GRIN3A, GABRA5
Neuronal System R-HSA−112316	Reactome 2022	0.0003254	GABRA3, GLRA3, GABRA2, GABRA5, GABRB1, RASGRF1, LRRC7, GABBR2, CACNG3, GRIN3A, SLC6A1
GABAergic synapse	KEGG 2021 Human	0.0002121	GABRA5, SLC6A1, GABBR2, GABRB1, GABRA3, GABRA2
GABA Receptor Activation R-HSA−977443	Reactome 2022	0.0007622	GABRA2, GABRB1, GABRA5, GABRA3, GABBR2
Extracellular Matrix Organization R-HSA−1474244	Reactome 2022	0.0008246	FN1, MMP2, COL2A1, COL22A1, KDR, BCAN, SERPINE1, TNR, FBN2
Focal adhesion	KEGG 2021 Human	0.001514	TNR, COL2A1, TNC, SPP1, FN1, KDR, RASGRF1
PI3K-Akt signaling pathway	KEGG 2021 Human	0.001514	TNR, IL7R, KDR, COL2A1, SPP1, TLR4, FN1, EPHA2, TNC
ECM-receptor interaction	KEGG 2021 Human	0.001514	FN1, TNR, SPP1, TNC, COL2A1
Morphine addiction	KEGG 2021 Human	0.001522	GABRA3, GABRA5, GABRB1, GABRA2, GABBR2
PrefrontalCortex	Human Gene Atlas	0.01486	CHD5, AQP4, LNX1, PTPRT, CALY, SNCB, GABRB1, GABBR2, MT3, BCAN
Signal Transduction R-HSA−162582	Reactome 2022	0.01658	SUCNR1, CNTN1, RASGRF1, SERPINE1, MMP2, HTR5A, GABBR2, TAC1, FN1, SOX6, FGFBP2, CDKN2B, NR5A2, PTHLH, GABRB1, FST, PNOC, PLPPR4, EPHA2, ARHGAP20, LRRC7, GFAP, KDR, SSTR1, MATK
Retrograde endocannabinoid signaling	KEGG 2021 Human	0.01156	GABRA2, GABRB1, GABRA5, GABRA3, SLC17A8
Taste transduction	KEGG 2021 Human	0.01156	GABRA3, GABBR2, GABRA2, GABRA5
Wholebrain	Human Gene Atlas	0.03463	LNX1, GABRA5, LRRC7, CACNG3, MT3, GABBR2
AGE-RAGE signaling pathway in diabetic complications	KEGG 2021 Human	0.01822	AGT, FN1, SERPINE1, MMP2

within the Human Gene Atlas: Prefrontal Cortex (q < 0.02) and Wholebrain (q < 0.04). Ten genes that are upregulated in the prefrontal cortex were found *(AQP4, BCAN, CALY, GABBR2, GABRB1, LNX1, MT3, PTPRT, SNCB,* and *CHD5*), together with six genes upregulated in the whole brain (CACNG3, GABBR2, GABRA5, LNX1, LRRC7, and MT3). These results are in line with the expected generation of forebrain-type neurons, using the STEMdiff™ Forebrain Neuron Kits, since the prefrontal cortex is part of the forebrain. Six pathways were significantly enriched using the Reactome dataset: Neurotransmitter Receptors and Postsynaptic Signal Transmission (ten genes, q < 0.000006), Transmission Across Chemical Synapses (eleven genes, q < 0.000006), Neuronal System (eleven genes, q < 0.0004), GABA Receptor Activation (five genes, q < 0.0007), Extracellular Matrix Organization (nine genes, q < 0.0008), and Signal Transduction (25 genes, q < 0.02). The top four pathways show a hierarchal relationship with the majority of genes recurring in all pathways and a greater number of genes being related to the neurotransmitter GABA, including *GABBR2, GABRA2, GABRA3, GABRA5, GABRB1, and SLC6A1* (Ghit et al., 2021). The Neuronal System constitutes the top hierarchy, which includes the events Transmission Across Chemical Synapses, Neurotransmitter Receptors and Postsynaptic Signal Transmission, and GABA Receptor Activation, in descending order. These results imply that the cell cultures are of neuronal type and, to a large extent, consist of GABAergic cells. The two remaining reactome enriched pathways, Extracellular Matrix Organization and Signal Transduction, are both driven by genes related to cell proliferation and differentiation (Kular et al., 2014, Watanabe and Osada, 2017) and were expected to be involved during the maturation of the cells. additionally, the PEA using KEGG identified GABA-related pathway enrichments, including Nicotine addiction, GABAergic synapse, Morphine addiction, Retrograde endocannabinoid signaling, and Taste transduction. Also, the enrichment of the pathway Neuroactive ligand-receptor interaction is mainly driven by genes encoding GABA-related proteins and also genes involved in other neuronal transmission activities, including *HTR5A, TAC1*, SSTR1, *GLRA3*, and *GRIN3A*. The genes, including *COL2A1, FN1, SPP1, TNC,* and TNR, mainly drive the pathways of Focal adhesion, PI3K-Akt signaling pathway, and ECM-receptor interaction. These pathways are all involved in cell survival, proliferation, and differentiation. The common genes are also related to cell migration and adhesion (Tsai et al., 2014, Viana et al., 2012). The enrichment of the last pathway, the AGE-RAGE signaling pathway in diabetic conditions, is driven by the genes *SERPINE1, FN1*, *MMP2*, and AGT. The former three are related to the composition of the extracellular matrix, while AGT is expressed in astrocytes (Sherrod et al., 2005), and the increased expression, in this case, may reflect the rising number of astrocytes during the culture period.

## 4 Discussion

Several successful *in vitro* studies have been previously performed to characterize human neural stem cells derived from human embryonic stem cells (Li et al., 2017, Heikkilä et al., 2009, Park et al., 2005, van de Leemput et al., 2014). Some of these have employed either feeder layers or aggregate-based culture systems. While aggregate cultures more closely mimic in vivo conditions (Salehi et al., 2020), 2D monolayer cultures are more practical for experimental manipulation and analysis. In a previous study, RNA sequencing was employed to analyze neuronal development over a 10-day differentiation culture (de Leeuw et al., 2022, de Leeuw et al., 2020). Their findings suggested that more culture time may be needed for the stronger expression of astroglial markers. Our temporal analysis of maturing neurons supports this notion, as we observed a proportional increase in the expression of markers such as *S100β, GFAP, and SLC1A3*. The increasing expression of these markers over the culture period, along with neuronal markers (e.g., *MAP2, DLG4*, and *SYP*), suggests that this culture is an astrocyte-neuronal co-culture. *DCX,* a marker associated with neuronal migration (Yap et al., 2016, Zare et al., 2019), showed increased expression and sustained presence in our 2D culture, consistent with previous observations in iPSC-derived neuronal differentiation (2D and 3D) culture systems (Lislien et al., 2025) and may reflect ongoing neurogenesis (Couillard-Despres et al., 2005).

We have identified the top 100 genes that drive the clustering of cells during neuronal maturation in this culture. Moreover, we have identified pathways that are enriched based on these genes. As expected, the regulation of several of them, such as *ROBO3*, *NeuroD6, CNTN1*, *LPPR4,* and *KIF21B*, is associated with neurogenesis, neuronal migration, synapse formation, and axon development (Cariboni et al., 2012, Uittenbogaard et al., 2010, Haenisch et al., 2005, Alvarez et al., 2023, Yu et al., 2015). Several of the enriched pathways using both KEGG and Reactome datasets further implicate this. The majority of the enriched pathways constitute neuronal-related processes such as neurotransmission (e.g., Neurotransmitter receptors and signal transmission, and transmission across chemical synapses) or receptors associated such as neuroactive ligand-receptor interaction and GABA receptor activation.

We have also identified the top 20 differentially expressed genes from each maturation time point vs day 0. Among them are genes that are associated with different stages of nervous system development, such as *Neurod6, NRN1, LHX2,* and *DMBX1*. Among the top 20 genes identified in our study is *C1Orf61* (CROC-4), which a previous study has discussed for its possible role in brain development (Jeffrey et al., 2000); therefore, its presence in our top genes may support their finding. Several other genes, such as *TNC, GRIN1,* and *PTGDS*, are also associated with neuronal maturation, migration, synaptic function, and axogenesis (Cariboni et al., 2012, Uittenbogaard et al., 2010, Tucić et al., 2021). Among them, some genes are astroglial markers, such as *GFAP* and *AQP4*.

Apart from the top 100 and top 20 genes that were identified using the unbiased bioinformatic tools, we also looked into the expression levels of several classical genes that are being investigated when it comes to neurodevelopmental studies, particularly *in vitro,* to monitor the various stages of the culture system using RT-qPCR, immunofluorescence, western blotting or whole transcriptome analysis. To achieve this, we used our data from Ion AmpliSeq™ to analyze selected markers specific for neuroepithelial, radial glial, immature neurons, and mature neurons. As shown in the result section, neuroepithelial markers (*Hes1* and *Sox2*) were downregulated compared to day 0. On the other hand, the initial increase of *Pax 6*, *Nes*, and *Hes5* as radial glial markers, followed by subsequent downregulation, suggests the emergence of radial glial cells at five days post-maturation. A similar expression trend for *Nes* was also observed in the study by Červenka et al. (2021). According to them, they did not observe a significant change in *Sox2* expression during the differentiation period, while our culture showed a significant decrease of this marker. They also mentioned an initial decrease in *S100B* expression followed by a later increase. Similarly, in our study, we observed a non-significant reduction at day 5, with levels rising after day 10 and peaking by day 30.

The continuously increased expression of markers for mature neurons, *MAP2, DLG4, and SYP*, also follows the expected trend of neuronal development and confirms the importance of the culture period to achieve more mature neurons. Moreover, several of the discussed genes showed a significant change in expression as early as day 5, suggesting that the induction of maturation has rapidly and significantly affected the fate of the culture. Hence, although we believe a longer culture will give a better-defined neuronal-astrocyte co-culture, a shorter maturation time could also be enough for quick downstream analysis, depending on the specific study objectives.

An interesting finding of this analysis is that several GABA-associated genes are among the genes driving the clustering of cells during maturation. These genes include *GABBR2, GABRB1, GABRA2, GABRA5, GABRA3,* and *SLC6A1* (also known as *GAT-1*, which is also in the top 20 differentially expressed genes). At least 2 or 3 of them are common in all of the enriched pathways. These results suggest that this astrocyte-neuron co-culture is strongly fated toward GABAergic neurons, consistent with previous characterization reports (Lislien et al., 2025, Pistollato et al., 2017). Although a tendency toward a glutamatergic fate has also been reported (Li et al., 2017, van de Leemput et al., 2014, Lislien et al., 2025). GABAergic interneurons are distributed throughout various regions of the brain, where they play a key role in inhibitory signaling and modulating neural circuits. With increasing evidence linking disruptions or imbalances in their activity to the onset of neurodevelopmental conditions such as Autism spectrum disorder (ASD) and related disorders (van de Leemput et al., 2014, LeBlanc and Fagiolini, 2011, Di et al., 2020, Tang et al., 2021). This culture system could be useful for studying the effects of compounds that influence, or are expected to influence, GABAergic neuron development. While this particular finding was more of a discovery than an intended outcome, there are already established protocols for differentiating GABAergic neurons from human stem cells in the literature using genetic factors (Liu et al., 2013, Sun et al., 2016, Gonzalez-Ramos et al., 2021, Maroof et al., 2013). However, unlike several studies where *GFAP* expression was minimal in differentiated neurons (Sun et al., 2016, Gonzalez-Ramos et al., 2021), by day 30, our culture exhibited high *GFAP* expression, ranking among the top identified genes. This finding is crucial for neurodevelopmental *in vitro* screening. Co-culture systems are particularly valuable in this context, as they replicate neuron-astrocyte interactions, better mimicking the *in vivo* microenvironment (De Simone et al., 2017; Enright et al., 2020, Attoff et al., 2017).

The culture medium employed in this study is a commercial kit, claiming to yield forebrain-specific neurons. However, our data did not reveal the presence of the *FOXG1* transcript, which is a classical marker for forebrain neurons. Our neural stem cells were maintained in the forebrain differentiation medium until reaching confluency, which typically took 2–3 days. Subsequently, they were plated in the forebrain maturation medium. The kit's timeline differed slightly, as it recommended keeping the neural stem cells for longer days in the forebrain differentiation medium until reaching confluency before inducing maturation. Hence, we cannot exclusively attribute the absence of the *FOXG1* transcript to this duration, but we cannot definitively reject it either. Based on our analysis, there are expressions of genes associated with forebrain development, such as Pax6 (Georgala et al., 2011) and *DLX1 and DLX2*, which are among the genes driving the variance and are associated with the development of forebrain GABAergic neurons (Lindtner et al., 2019). In particular, *DLX2* is one of the transcription factors that have been used previously to induce GABAergic neurons (Gonzalez-Ramos et al., 2021, Yang et al., 2017). Additionally, with the presence of the prefrontal cortex, also observed by van de Leemput et al. (2014) as one of the enriched cell types based on the Human Gene Atlas dataset, we still acknowledge that our cultures may not be entirely forebrain-specific in all aspects. It is important to note that in this study, we employed the Ion AmpliSeq Transcriptome Panel, which targets approximately 20,000 genes. This number aligns with current consensus estimates of human protein-coding genes; for instance, reports around 19,955 protein-coding genes (Frankish et al., 2019). While some estimates suggest that the total number of protein-coding genes could be as high as 25,000 (International Human Genome Sequencing Consortium, 2004), our panel is based on widely accepted annotations that approximate the number to around 20,000. Although the panel is widely validated and commonly used, its design, based on current genome annotations, may limit the detection of transcripts not included in the panel, potentially overlooking novel or unannotated genes.

## 5 Conclusion and future perspectives

In this study, the aim was to investigate the transcript profile of maturing neurons differentiated from human neural stem cells that were successfully derived from H9 embryonic stem cells in our previous study without the use of mouse embryonic fibroblasts (Hosseini et al., 2020). Our findings support prior studies, confirming that these neuronal cultures are robust, reproducible, and suitable for high-throughput screening setups. Using 4-5 independent replicates, each from a different passage, we observed consistent gene expression trends, with all replicates following the expected maturation trajectory. Transcriptomic analysis, along with PEA, revealed a predominance of GABAergic phenotypes, supported by the top 100 genes, alongside a progressive increase in astrocyte markers over time. It is important to note that certain genes are expressed in both neurons and other neural cell types, and their expression levels can vary significantly in response to surrounding stimuli. Since our analysis focused solely on transcriptional data, further investigation can validate these findings. For example, employing flow cytometry with cell-specific markers for neurons, astrocytes, oligodendrocytes, and neural stem cells could provide a quantitative assessment of the culture's purity, increasing the reliability of downstream analyses. Immunostaining and functional assays, such as electrophysiology, would offer complementary insights into protein expression and cellular function. Additionally, single-cell analysis could deepen our understanding of the culture's heterogeneity and how individual cell types respond to different environmental cues—a particularly important consideration when studying the neuro-developmental impact of specific compounds.

## Author contribution

K.H.: Designed the research, performed cell culture, immunocytochemistry, RNA extraction, analyzed the results, and wrote the manuscript. G.P.: Designed the research, performed RNA extraction, and edited the manuscript. S.B.S.: Analyzed data and wrote part of the manuscript. A.C.: Analyzed the staining images using CellProfiler and wrote part of the manuscript. E.GH.: Performed immunocytochemistry. R.F.: Designed the research, analyzed the data, wrote part of the manuscript, and funded the research.

## CRediT authorship contribution statement

**Kimia Hosseini:** Writing – original draft, Visualization, Methodology, Formal analysis, Conceptualization. **Gaëtan Philippot:** Writing – review & editing, Methodology, Conceptualization. **Sara B. Salomonsson:** Writing – original draft, Software, Formal analysis, Data curation. **Andrea Cediel-Ulloa:** Writing – review & editing, Formal analysis. **Elnaz Gholizadeh:** Writing – review & editing, Methodology. **Robert Fredriksson:** Writing – review & editing, Supervision, Funding acquisition, Formal analysis, Data curation, Conceptualization.

## Declaration of Competing Interest

The authors declare that they have no known competing financial interests or personal relationships that could have appeared to influence the work reported in this paper. The authors declare no competing interest.
