# Supplementary material for: Transcriptomic characterization of maturing neurons from human neural stem cells across developmental time points
Source: IBRO Neurosci Rep. 2025 Apr 17;18:679–89. doi: 10.1016/j.ibneur.2025.04.013 (PMC12056963; doi:10.1016/j.ibneur.2025.04.013)
Supplement: Supplementary file 1 — Supplementary material [file mmc1.docx]

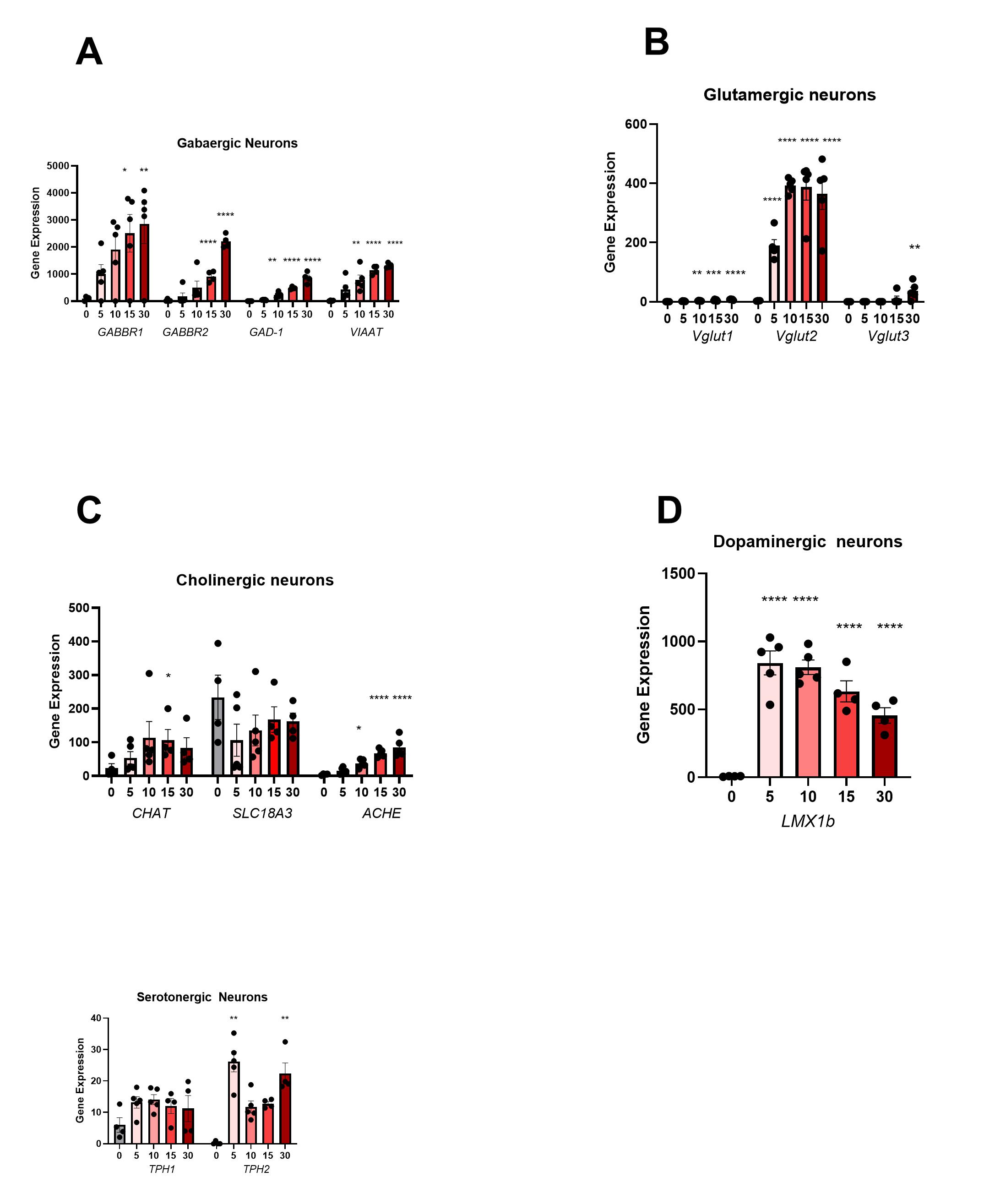


Supplementary Figure 1: **Temporal expression of selectively chosen neuronal subtype markers at different time points.** A. GABAergic Neurons: Expression of GABAergic markers increases over time, with a steady rise in gene expression from day 0 to day 30. B. Glutamatergic Neurons: SLC17A6 shows the highest expression among the glutamatergic markers, with SLC17A7 and SLC17A8 showing a modest but significant increase, though they remain at much lower levels compared to SLC17A6. C. Cholinergic Neurons: Cholinergic markers are present but at lower levels. with ACHE expression significantly increasing by day 10 and continuing to rise by day 30, though still lower than GABAergic marker levels. D. Dopaminergic Neurons: Dopaminergic markers are either absent, negligibly expressed, or, after an initial increase, show a downregulation over time. E. Serotonergic Neurons: Similar to dopaminergic neurons, they are mostly absent. TPH1 and TPH2 are expressed but at much lower level.
